# A Case of Recurrent Right-Sided Loculated Pleural Effusion With Right Psoas Abscess

**DOI:** 10.7759/cureus.20022

**Published:** 2021-11-29

**Authors:** Subodh Kumar, Aroop Mohanty, Vivek Hada, Gaurav Gupta, Shashank Sekhar

**Affiliations:** 1 Pulmonary Medicine, All India Institute of Medical Sciences (AIIMS) Gorakhpur, Gorakhpur, IND; 2 Clinical Microbiology, All India Institute of Medical Sciences (AIIMS) Gorakhpur, Gorakhpur, IND; 3 General Surgery, All India Institute of Medical Sciences (AIIMS) Gorakhpur, Gorakhpur, IND; 4 Radiotherapy, All India Institute of Medical Sciences (AIIMS) Gorakhpur, Gorakhpur, IND

**Keywords:** psoas abscess, truenat, thoracolumbar fascia, loculated pleural effusion, tuberculosis

## Abstract

Tuberculous psoas abscess is a rare entity and is mostly associated with tuberculosis of the spine in view of the close vicinity. It is mostly secondary to direct extension from adjacent structures. Here we present a case of a young man who presented to us with a persistent swelling in the right side of the back and with a history of taking anti-tubercular drugs for six months. He was finally diagnosed with a recurrent right-sided loculated pleural effusion with a right-sided psoas abscess. Surgical drainage of the psoas abscess was done and he was again started on anti-tubercular therapy (ATT).

## Introduction

Psoas (or Iliopsoas) abscess is a collection of pus in the iliopsoas compartment which is formed by the iliacus and psoas muscles. It is an extremely rare condition like many other extra-pulmonary forms of tuberculosis but with the improvements in the diagnostic techniques, its incidence has increased lately [1, 2]. It has been reported worldwide with about 12 new cases per year [3]. Iliopsoas abscess can be classified as primary or secondary. Primary abscess develops due to haematogenous spread of a causative organism from a distant site and is usually monomicrobial in nature whereas secondary abscess occurs due to direct expansion from a nearby infectious process being polymicrobial in nature [4]. In India majority of the cases are primary in origin with Mycobacterium tuberculosis being the commonest aetiology. Due to its vague presentation and non-specific symptomatology, it is associated with diagnostic delays and high morbidity. Here we report a case of psoas abscess secondary to a recurrent right-sided loculated pleural effusion due to Mycobacterium tuberculosis in a young male.

## Case presentation

A 21-year-old male patient presented to the Pulmonary Medicine OPD of a tertiary care centre in Eastern Uttar Pradesh with complaints of swelling over the posterior part of the right chest for the last two weeks. It was associated with on and off fever and decrease in appetite. He had a similar swelling on the same site two years back for which local incision and drainage was performed at a district hospital which provided him with symptomatic relief. One year later, he again developed the swelling over the same place, from which pus was aspirated after local incision and drainage. The pus sample was sent for MTB detection by GeneXpert. It detected Mycobacterium tuberculosis (MTB) complex which was sensitive to Rifampicin. He was immediately started on ATT (2HRZE + 4HRE) and he took it for six months with full compliance.

**Figure 1:**
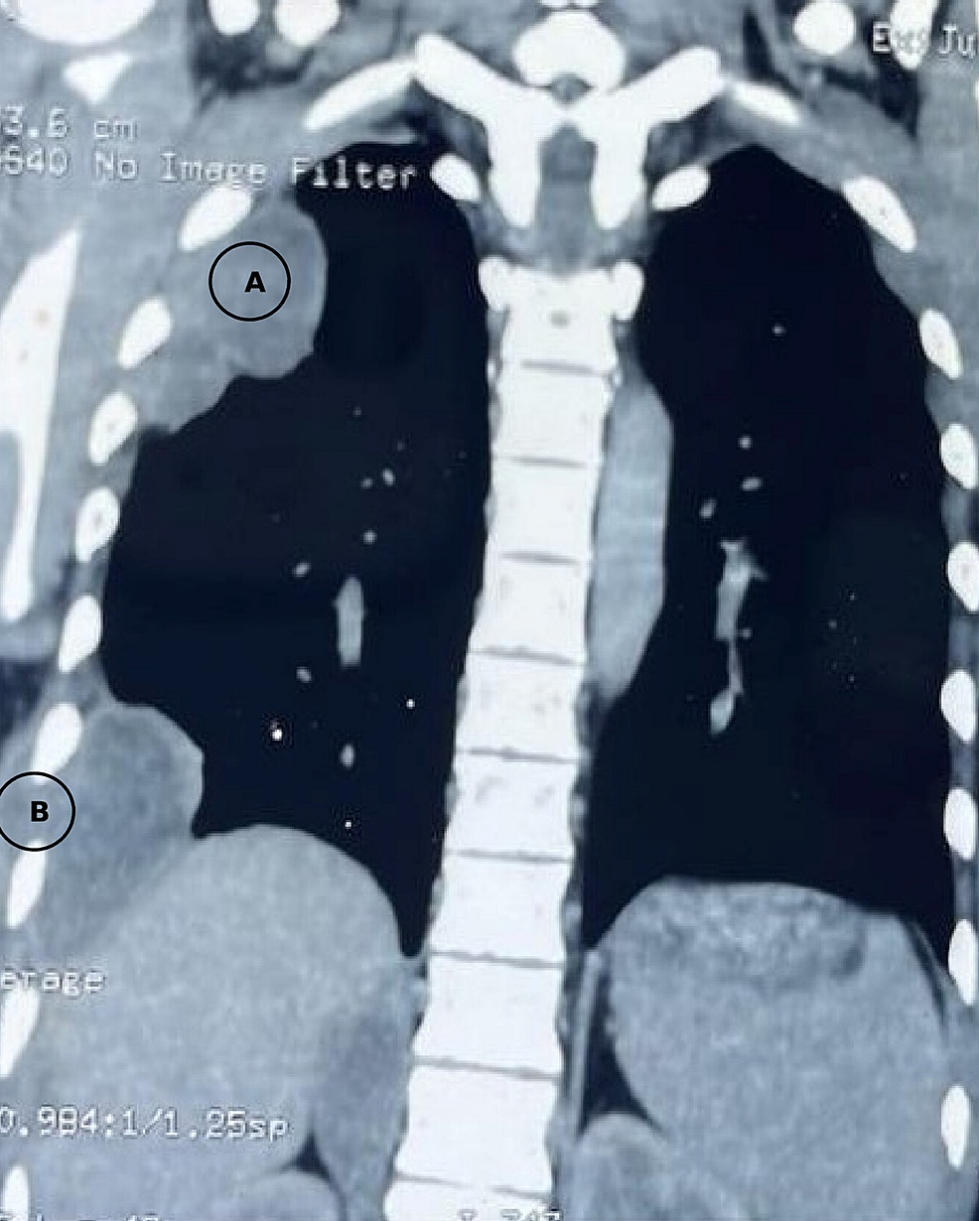
Contrast-enhanced computed tomography (CECT) thorax showing loculated pleural effusion (A) and pleural effusion with communication to subcutaneous plane (B).

On general examination in our OPD, he was found to be afebrile with heart rate of 80 beats/min, respiratory rate 20/min, blood pressure 118/76 mm Hg and oxygen saturation at 98% while breathing on room air. On local examination, the swelling was seen over the right paraspinal region, 10 x 10 cm in dimension and with fluctuation. It was associated with mild tenderness. An X-ray chest was done which revealed loculated pleural effusion. Further, a contrast-enhanced computed tomography (CECT) thorax was advised which showed an encysted pleural effusion on the right apical and lower lobe region and communicating to the subcutaneous plane (Figure 1). Pleural fluid was drained and sent to the Microbiology laboratory for detailed investigations. Gram stain of the sample revealed plenty of pus cells with no microorganisms. Acid-fast bacilli were not seen on Ziehl-Neelsen staining of the sample, but True Nat (Molbio Diagnostics, Goa, India) detected Rifampicin sensitive Mycobacterium tuberculosis. The patient was again initiated on ATT (2HRZE + 4HRE).

**Figure 2:**
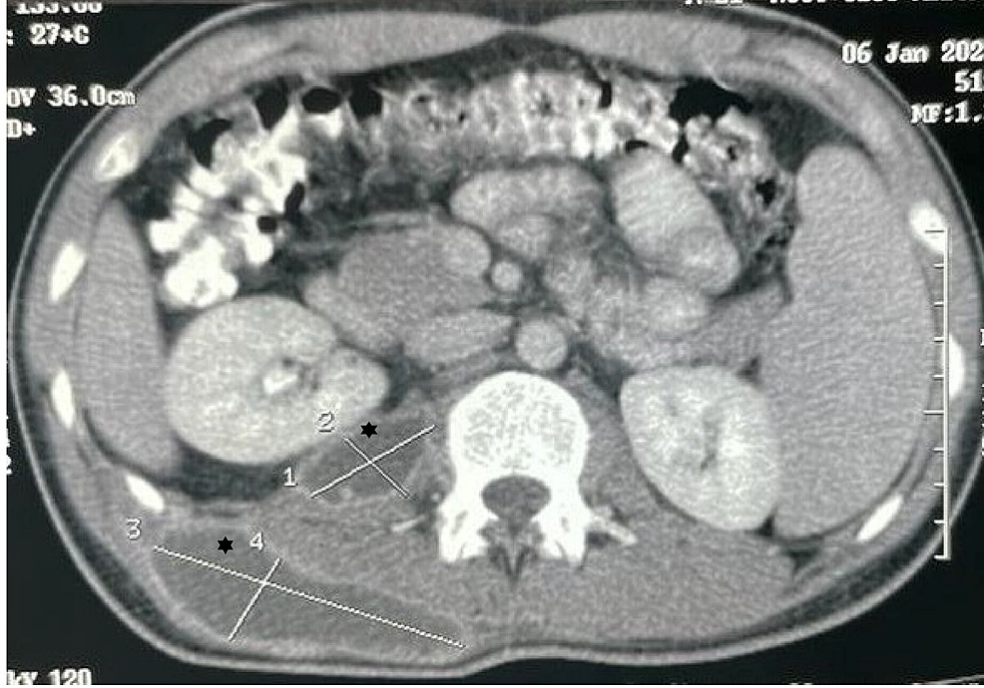
Contrast-enhanced computed tomography (CECT) abdomen showing abscess in psoas major and quadratus lumborum muscles.

Ultrasonography (USG) of the soft tissue swelling on the posterior part of the right chest wall was done in order to ascertain the extent of swelling. It showed a right psoas abscess tracking along the course of psoas muscle with breach in the posterior abdominal wall communicating with a collection in the subcutaneous plane at the posterior aspect of the right side of the chest. This was confirmed by a CECT whole abdomen which gave an impression of a well-defined non-enhancing cystic lesion in the right psoas muscle with extension into the right posterior abdominal wall with absence of spinal disease (Figure 2). No spinal finally, the patient was diagnosed as a case of recurrent right-sided loculated pleural effusion with right-sided psoas abscess. Drainage of the psoas abscess was done under local anaesthesia. The patient was continued on ATT and advised to come for follow-up after four weeks. On follow-up visit, he was symptomatically better with a considerable reduction in the size of the swelling.

## Discussion

The iliopsoas compartment is an extraperitoneal space containing the iliopsoas and iliacus muscle. This space is defined by the psoas fascia and is a direct communication from the mediastinum to the thigh. Psoas abscess was initially described as psoitis and is responsible for 5-10% of all abdominal suppurations [5].

It may be classified as primary or secondary depending on the presence or absence of an underlying aetiology. Majority of them are secondary in nature (70%). The primary ones have an underlying cause and usually spread via haematogenous or lymphatic spread especially in immunosuppressed conditions like diabetes mellitus, renal failure, HIV, intravenous drug abuse and other chronic diseases. In contrast, secondary abscesses result from local extension from an infective process. Owing to the close proximity to organs like appendix, colon, kidneys, ureters, iliac lymph nodes and spine, any underlying disease in these organs can spread to the iliopsoas muscle and result in psoas abscess. In a study comprising 367 cases, Ricci et al. noted that Crohn’s disease was the commonest cause of secondary psoas abscess [6]. This is quite different in developing countries where mycobacterial psoas abscess is typically associated with spinal tuberculosis due to the extension of infection from the lumbar spine. However, as in our case, a psoas abscess may occur in the absence of spinal disease. Here it was secondary to the right-sided recurrent loculated tubercular pleural effusion. It was possibly missed in the initial course of the disease and was never even looked for. The infection which was in form of a loculated effusion might have drained along the endothoracic fascia and then via the transversalis fascia to the thoracolumbar fascia [7]. The thoracolumbar fascia also known as lumbodorsal fascia is a deep investing membrane comprising of three layers with the anterior layer being the thinnest and posterior layer being the thickest. It is postulated that in our case the pus penetrated through the anterior and middle layers to accumulate in the psoas major and the adjoining areas leading to a psoas abscess.

Abdominal CT is the principal imaging modality for the diagnosis of psoas abscess, but as in our case USG provided us with the initial suspicion of the condition [8]. USG is very useful for drain insertion and also for monitoring resolution. A combination of radiological imaging along with confirmation by microbiological tests led to the final diagnosis of this condition.

## Conclusions

Psoas abscess in our country is mostly associated with tuberculosis of the spine. As majority of the psoas abscess cases are in good medical condition, it generally leads to late diagnosis, and patients usually present with chronic symptoms. It is very rarely seen in association with other adjoining infective process. This case, therefore, signifies the need of a thorough examination of patients with extra-pulmonary tuberculosis along with evaluation of other multiple possible locations.
